# A SnO_2_/ZnO Nanoparticle Bilayer Electron Transport Layer for Regulated Electron Injection in Quantum-Dot Light-Emitting Diodes

**DOI:** 10.3390/nano16161003

**Published:** 2026-08-15

**Authors:** Yuechao Wang, Xiongqiang Ma, Ruirong Wang, Junsheng Zhang

**Affiliations:** 1 Department of Electronic Engineering, Taiyuan Institute of Technology, Taiyuan 030008, China; wangrr@tit.edu.cn (R.W.); zhangjs@tit.edu.cn (J.Z.); 2 Department of Mining Engineering, Shanxi Institute of Energy, Taiyuan 030006, China; maxq@sxie.edu.cn

**Keywords:** QLEDs, SnO_2_/ZnO, bilayer electron transport layer, electron injection, leakage current

## Abstract

ZnO is widely used as an electron transport layer in quantum-dot light-emitting diodes (QLEDs) because of its high electron mobility and suitable energy levels. However, rapid electron transport may cause excessive electron injection, leading to charge accumulation and parasitic recombination. SnO_2_ provides weaker electron transport, but using it alone limits device performance. Here, a SnO_2_/ZnO bilayer electron transport layer was introduced to regulate electron injection. Unlike previously reported structures in which SnO_2_ directly contacts the QDs, the present configuration places SnO_2_ on ITO and ZnO adjacent to the QD layer. The low-concentration ZnO overlayer reduced the RMS roughness of the SnO_2_ film from 1.79 to 1.07 nm and facilitated electron injection, while the underlying SnO_2_ layer moderated the electron supply. The bilayer also suppressed leakage current and showed the lowest capacitance peak, consistent with improved charge balance. The bilayer QLED achieved a maximum current efficiency of 13.40 cd/A and a maximum luminance of 33,210 cd/m^2^. Its current efficiency was 100% and 32.7% higher than those of the SnO_2_ and ZnO devices, respectively. These results demonstrate that the bilayer improves charge balance through facilitated electron injection and controlled electron supply.

## 1. Introduction

Quantum-dot light-emitting diodes (QLEDs) have attracted considerable attention because of their high color purity, high brightness, and solution processability [1,2,3,4,5,6,7,8,9,10,11]. Most inverted QLEDs use ZnO nanoparticles as the electron transport layer (ETL) [12,13,14,15,16,17]. ZnO provides efficient electron transport and favorable energy-level alignment with quantum dots (QDs) [13,14,15,16,17]. However, its strong electron-transport ability may cause excessive electron injection when hole injection is limited. This imbalance can lead to charge accumulation and nonradiative recombination in the QD layer [15,18,19,20]. SnO_2_ has been investigated as an alternative ETL because it shows weaker interfacial interactions with QDs [21,22,23,24,25]. However, solution-processed SnO_2_ nanoparticle films usually have lower conductivity and rougher surfaces than ZnO films [21,22,23]. These properties may limit electron injection and increase leakage current. Therefore, neither ZnO nor SnO_2_ alone provides well-regulated electron injection in the present QLED structure [10,26,27,28,29].

Bilayer ETLs offer a possible route to regulate electron injection. Double-ZnO and ZnMgO/ZnO structures have been used to improve charge balance in QLEDs [30,31,32]. SnO_2_/ZnO combinations have also been reported. Park et al. inserted a SnO_2_ layer between ZnO and QDs in an inverted QLED. The SnO_2_ layer suppressed spontaneous electron injection from ZnO and improved charge balance [33]. Ye et al. placed a SnO_2_ buffer layer directly adjacent to the QDs [34]. The SnO_2_ layer reduced interfacial quenching and stabilized the QD PL decay, while ZnO maintained electron transport. In both reported structures, SnO_2_ was placed adjacent to the QD layer and mainly served as an interfacial buffer. In contrast, the reverse configuration, with SnO_2_ deposited on ITO and ZnO directly contacting the QDs, has received less attention. In this configuration, the ZnO overlayer may improve the surface morphology of SnO_2_ and facilitate electron injection into the QD layer. The underlying SnO_2_ layer may, in turn, regulate electron transport through the bilayer. This configuration may suppress leakage and improve charge balance without strongly limiting electron transport.

In this work, inverted CdSe/ZnS QLEDs employing SnO_2_, ZnO, and reverse SnO_2_/ZnO ETLs were fabricated and compared. The relationship between surface morphology, electron transport, charge accumulation, and electroluminescence was systematically examined. AFM and *J–L–V* measurements were used to evaluate surface roughness and leakage current. Electron-only and *C–V* measurements were then employed to examine electron transport. AFM and TRPL measurements were further used to assess the morphology and PL decay of QD films on different ETLs. Finally, EL spectra and transient EL responses were used to examine spectral differences and transient carrier behavior. The results show that the ZnO overlayer smooths the SnO_2_ surface and maintains effective electron transport, whereas the bilayer exhibits electrical characteristics consistent with improved charge balance.

## 2. Materials and Methods

CdSe/ZnS quantum dots (QDs) were purchased from Najing Technology Co., Ltd. (Hangzhou, China) 4,4′-Bis(carbazol-9-yl)biphenyl (CBP) and molybdenum trioxide (MoO_3_) were purchased from Xi’an Polymer Light Technology Corp. (Xi’an, China) The commercial SnO_2_ dispersion was purchased from Alfa Aesar Chemical Co., Ltd. (Shanghai, China) and post-treated following our previously reported procedure [21]. ZnO nanoparticles were synthesized following our previously reported procedure [35]. The particle sizes of the SnO_2_ and ZnO nanoparticles were approximately 3–5 nm, as reported previously [21,35]. Ethanol was used as the solvent for both the SnO_2_ and ZnO dispersions.

**Figure 1:**
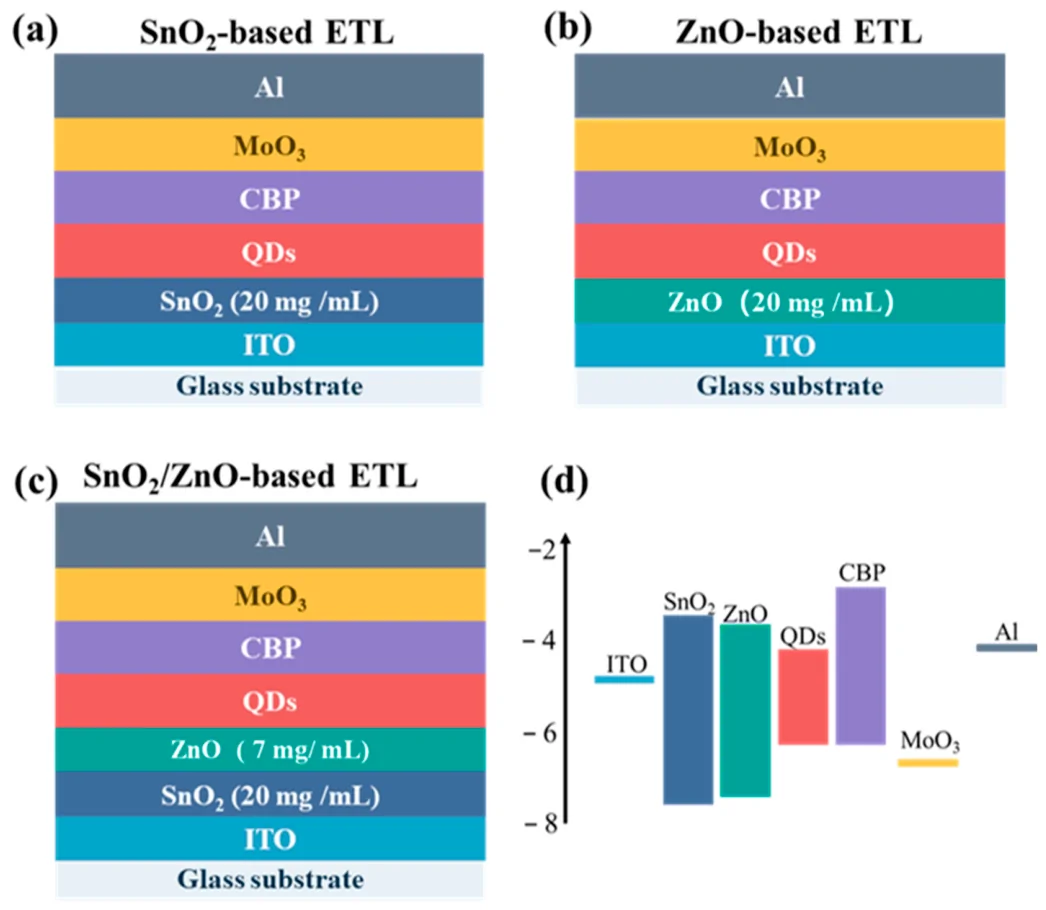
Device structures of inverted QLEDs employing (**a**) SnO_2_, (**b**) ZnO, and (**c**) SnO_2_/ZnO electron transport layers. (**d**) Schematic energy-level alignment of SnO_2_, ZnO, CdSe/ZnS QDs, and CBP.

Three QLED structures were fabricated: glass/ITO/SnO_2_/QDs/CBP/MoO_3_/Al, glass/ITO/ZnO/QDs/CBP/MoO_3_/Al, and glass/ITO/SnO_2_/ZnO/QDs/CBP/MoO_3_/Al, as shown in Figure 1a–c. Figure 1d presents a literature-based schematic energy-level alignment of SnO_2_, ZnO, CdSe/ZnS QDs, and CBP. The diagram was constructed using values reported previously [22,35]. In the single-layer devices, the concentrations of the ZnO and SnO_2_ solutions were both 20 mg/mL. For the bilayer device, the concentrations of the SnO_2_ and ZnO solutions were 20 and 7 mg/mL, respectively. A reverse-order control device with the structure glass/ITO/ZnO/SnO_2_/QDs/CBP/MoO_3_/Al was also prepared using 20 mg/mL ZnO and 7 mg/mL SnO_2._ A process-matched SnO_2_–solvent control with the structure glass/ITO/SnO_2_–solvent/QDs/CBP/MoO_3_/Al was prepared to reproduce the solvent exposure and second annealing used for the bilayer device.

The patterned ITO substrates were sequentially cleaned with acetone, ethanol, and deionized water by ultrasonication for 15 min in each solvent. The substrates were then dried with nitrogen and treated with UV–ozone for 5 min. All solution-processing steps were carried out in a nitrogen-filled glovebox. For the single-layer ZnO device, a 20 mg/mL ZnO solution was spin-coated onto ITO at 2000 rpm for 60 s and annealed at 120 °C for 30 min. For the single-layer SnO_2_ device, a 20 mg/mL SnO_2_ solution was spin-coated at 2000 rpm for 60 s and annealed at 150 °C for 15 min. The same SnO_2_ deposition process was used for the bilayer device. A 7 mg/mL ZnO solution was then deposited onto the SnO_2_ layer at 2000 rpm for 60 s and annealed at 120 °C for 30 min. For the reverse-order control, the 20 mg/mL ZnO layer was prepared as described above. A 7 mg/mL SnO_2_ dispersion was then spin-coated at 2000 rpm for 60 s and annealed at 150 °C for 15 min. The process-matched SnO_2_–solvent control was prepared by spin-coating the solvent used for the ZnO dispersion (ethanol) onto the annealed SnO_2_ film at 2000 rpm for 60 s, followed by annealing at 120 °C for 30 min. The solvent evaporated during this annealing step. For AFM and TRPL measurements, QD films were deposited on glass/ITO/SnO_2_, glass/ITO/ZnO, and glass/ITO/SnO_2_/ZnO under the same conditions used for the QLEDs. A glass/QD sample was also prepared as the substrate reference for TRPL.

The QD solution (12 mg/mL) was spin-coated onto the electron-transport layer at 2000 rpm for 60 s, followed by annealing at 80 °C for 30 min. The substrates were then transferred into a thermal evaporation chamber. CBP (60 nm), MoO_3_ (8 nm), and Al (100 nm) were sequentially deposited under a vacuum below 4 × 10^−4^ Pa. The deposition rates were 0.03–0.05 nm/s for CBP, 0.01–0.03 nm/s for MoO_3_, and 0.25 nm/s for Al. The active emitting area of each QLED was 2 mm × 2 mm.

Electron-only devices were prepared with the structures glass/ITO/SnO_2_/QDs/SnO_2_/Al, glass/ITO/ZnO/QDs/ZnO/Al, and glass/ITO/SnO_2_/ZnO/QDs/ZnO/SnO_2_/Al. A hole-only device with the structure ITO/CBP/MoO_3_/Al was also prepared for comparison. All layers were prepared under the same conditions as those used in the corresponding QLEDs.

The current density–voltage–luminance characteristics were measured using a Keithley 2400 source meter (Keithley Instruments, Cleveland, OH, USA) and a calibrated SpectraScan PR-655 spectroradiometer (Photo Research, Inc., Chatsworth, CA, USA). Electroluminescence spectra were recorded using a Maya 2000 Pro spectrometer (Ocean Optics, Dunedin, FL, USA). The EL spectra were background-subtracted before analysis. The film morphologies and step heights were measured by AFM (CoreAFM, Nanosurf AG, Liestal, Switzerland). The capacitance–voltage characteristics were measured using a precision LCR meter (TH2829C, Changzhou Tonghui Electronic Co., Ltd., Changzhou, China) at a frequency of 1 kHz with an AC signal of 100 mV. The same instrument was used for the impedance–voltage measurements. Transient electroluminescence measurements were performed using a RIGOL DG5102 waveform generator (RIGOL Technologies Co., Ltd., Suzhou, China), a Zolix PMTH-S1-CR131 photodetector (Zolix Instruments Co., Ltd., Beijing, China), and a RIGOL DS4054 oscilloscope (RIGOL Technologies Co., Ltd., Suzhou, China). A square-wave pulse ranging from 0 to 5 V at a frequency of 1 kHz was applied to the devices. The UV–visible absorption spectra of the SnO_2_ and ZnO films were recorded with a UV-1750 spectrophotometer (Shimadzu Corporation, Kyoto, Japan). The time-resolved PL decays of the QD films were recorded with a QuantaMaster 8000 fluorescence spectrometer (HORIBA Scientific, Piscataway, NJ, USA). The experimental data were processed and plotted using Origin (OriginLab Corporation, Northampton, MA, USA). All QLEDs were encapsulated before measurement and characterized under ambient conditions.

## 3. Results and Discussion

**Figure 2:**
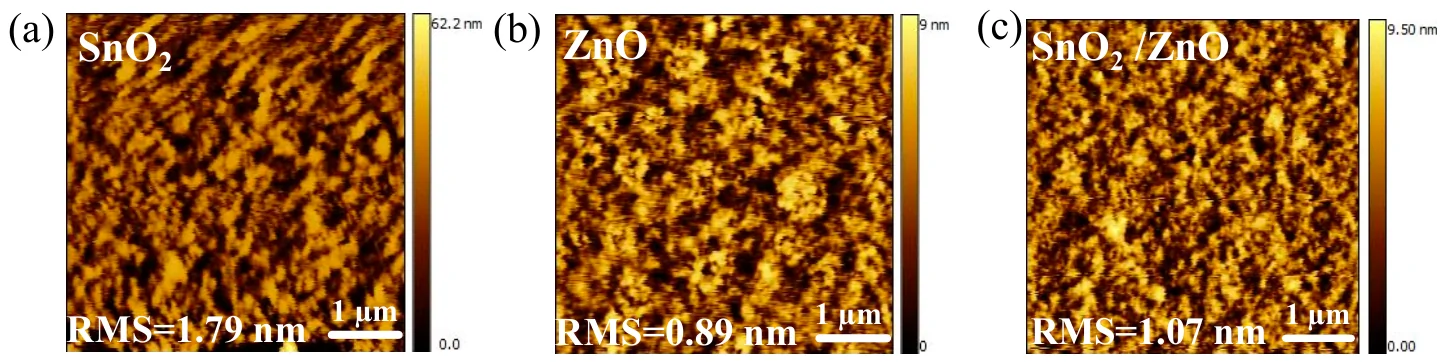
AFM images of (**a**) SnO_2_, (**b**) ZnO and (**c**) SnO_2_/ZnO nanoparticle films deposited on ITO substrates.

To clarify how the ZnO overlayer modifies the SnO_2_-based ETL, the surface morphologies of the SnO_2_, ZnO, and SnO_2_/ZnO films were first examined by AFM. As shown in Figure 2a–c, the SnO_2_ film exhibited a root-mean-square (RMS) roughness of 1.79 nm, whereas the ZnO film showed a lower value of 0.89 nm. After a thin ZnO layer was deposited on SnO_2_, the RMS roughness decreased to 1.07 nm. This corresponds to a reduction of approximately 40% compared with the single SnO_2_ film, and the resulting value is close to that of the ZnO film. These results indicate that the ZnO overlayer reduced the surface roughness of the SnO_2_-based ETL. The smoother surface is expected to provide a more favorable interface for the subsequent deposition of the QD layer and reduce the possibility of local leakage pathways.

The morphology of the QD films deposited on each ETL was also examined by AFM. As shown in Figure S1, the RMS roughness was 1.80 nm on SnO_2_, 1.32 nm on ZnO, and 1.41 nm on SnO_2_/ZnO. Thus, the QD film on SnO_2_/ZnO was smoother than that on SnO_2_ and comparable to that on ZnO. The AFM step-height profiles are shown in Figure S2. The SnO_2_, ZnO, SnO_2_/ZnO, and QD films were approximately 40, 40, 50, and 20 nm thick, respectively.

**Figure 3:**
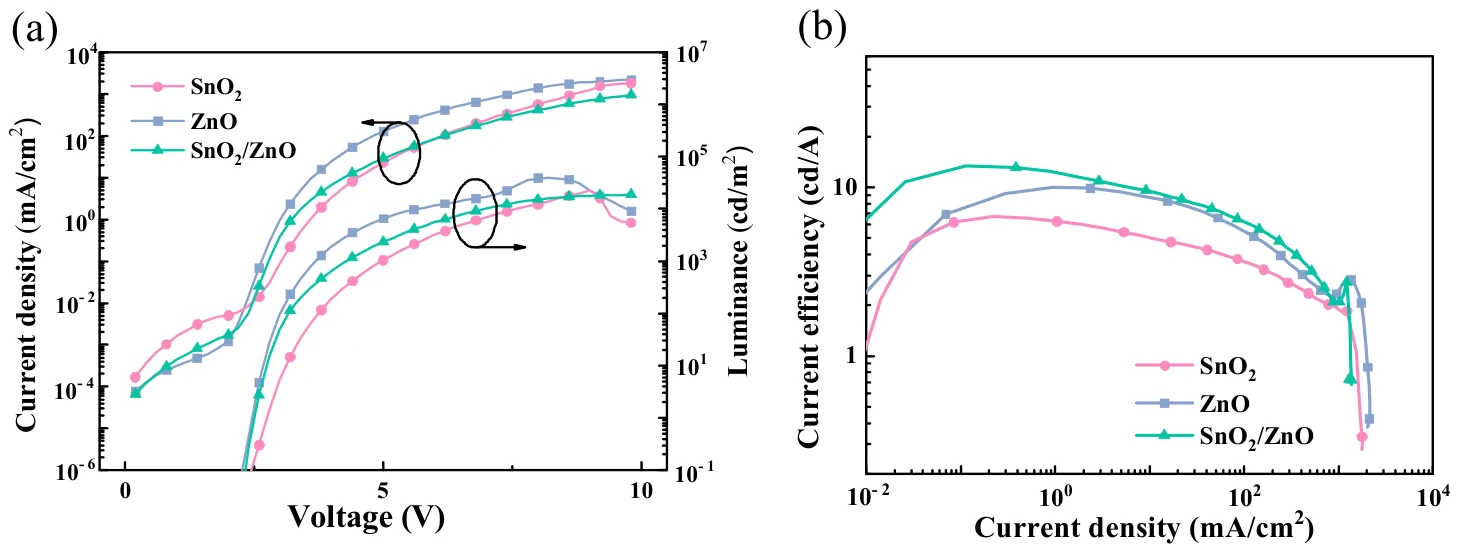
(**a**) *J–L–V* characteristics and (**b**) CE*–J* characteristics of QLEDs with different ETLs.

Following the surface morphology analysis, the influence of different ETLs on device performance was investigated. Figure 3a shows the *J-V* and *L-V* characteristics on the left and right y-axes, respectively. Before turn-on, the SnO_2_-based device exhibited the highest current density, indicating a larger leakage current. After a thin ZnO layer was deposited on SnO_2_, the current density before turn-on was reduced and became comparable to that of the ZnO-based device. This result is consistent with the reduced RMS roughness observed by AFM. It suggests that the smoother SnO_2_/ZnO surface decreases the possibility of local leakage pathways. To assess the effects of the solvent treatment and second annealing, a process-matched SnO_2_–solvent control was prepared. The control film had an RMS roughness of 1.64 nm, close to 1.79 nm for untreated SnO_2_, as shown in Figure S3a. Both devices showed similar *J–V*, *L–V*, and CE*–J* characteristics (Figure S3b–d). Thus, the solvent treatment and second annealing had little effect on the SnO_2_ film or device performance.

After the devices were turned on, clear differences in current density were observed. The ZnO-based device generally exhibited the highest current density, which is consistent with the stronger electron transport capability of ZnO. The current density of the SnO_2_/ZnO device was higher than that of the SnO_2_ device but remained lower than that of the ZnO device. Because the hole-side layers were identical in all three devices, these differences can be mainly associated with the electron injection and transport characteristics of the ETLs. The results suggest that the ZnO overlayer enhances electron transport in the SnO_2_-based device, while the underlying SnO_2_ layer moderates electron injection compared with the single ZnO ETL. The reverse-order ZnO/SnO_2_ device exhibited *J–V*, *L–V*, and CE*–J* characteristics similar to those of the single-layer ZnO device (Figure S4). No efficiency gain was observed. Thus, the bilayer improvement cannot be attributed solely to the presence of two oxides or the greater ETL thickness.

The corresponding *L-V* characteristics are also presented in Figure 3a. The turn-on voltage, defined at a luminance of 0.1 cd/m^2^, decreased from 2.5 V for the SnO_2_ device to 2.3 V for the SnO_2_/ZnO device. The latter value was identical to that of the ZnO device. The maximum luminance was 22,770 cd/m^2^ for the SnO_2_ device and 33,210 cd/m^2^ for the SnO_2_/ZnO device. This represents an increase of 45.9%. Although this value remained lower than the maximum luminance of the ZnO device (39,180 cd/m^2^), it confirms that the introduction of the ZnO layer improved the electroluminescence performance of the SnO_2_-based device.

**Figure 4:**
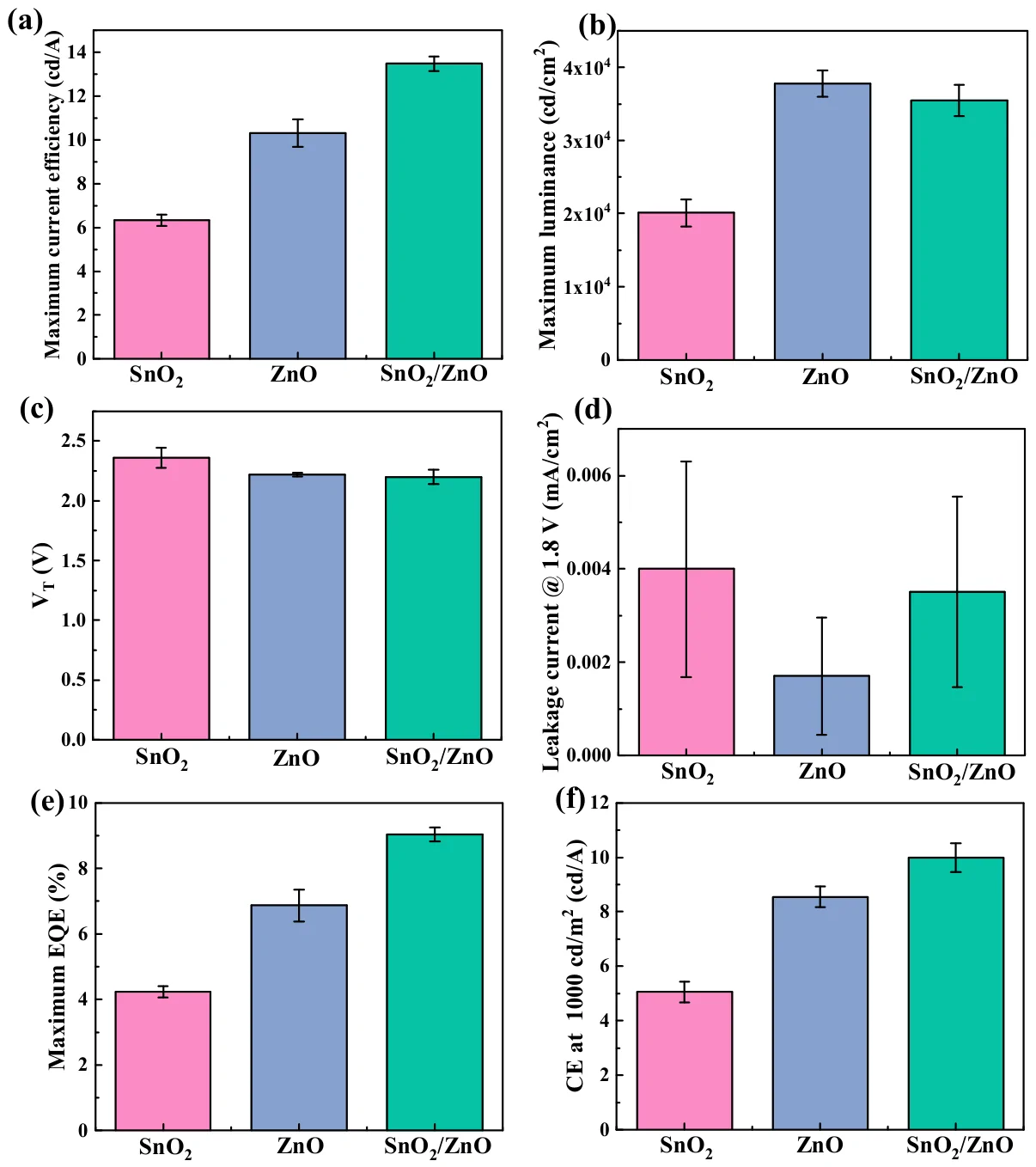
Statistical performance of QLEDs employing different ETLs: (**a**) maximum current efficiency, (**b**) maximum luminance, (**c**) turn-on voltage, (**d**) leakage current density at 1.8 V, (**e**) maximum EQE, and (**f**) current efficiency at 1000 cd/m^2^. The bars and error bars represent the mean values and standard deviations, respectively (*n* = 4). The individual device values are listed in Table S1.

Figure 3b shows the CE*–J* characteristics of the three devices. The SnO_2_/ZnO device achieved the maximum current efficiency of 13.40 cd/A. This value was 100% higher than that of the SnO_2_ device (6.70 cd/A) and 32.7% higher than that of the ZnO device (10.10 cd/A). Notably, the ZnO device exhibited the highest current density and maximum luminance, but its current efficiency was lower than that of the SnO_2_/ZnO device. This result indicates that a higher current density does not necessarily result in a higher current efficiency. The regulated electron injection provided by the SnO_2_/ZnO bilayer ETL, together with its reduced leakage current, may promote a more favorable balance between electrons and holes. For each ETL, four devices from independent fabrication batches were measured. Individual values and statistical results are listed in Table S1, and the corresponding means and standard deviations are shown in Figure 4.

**Figure 5:**
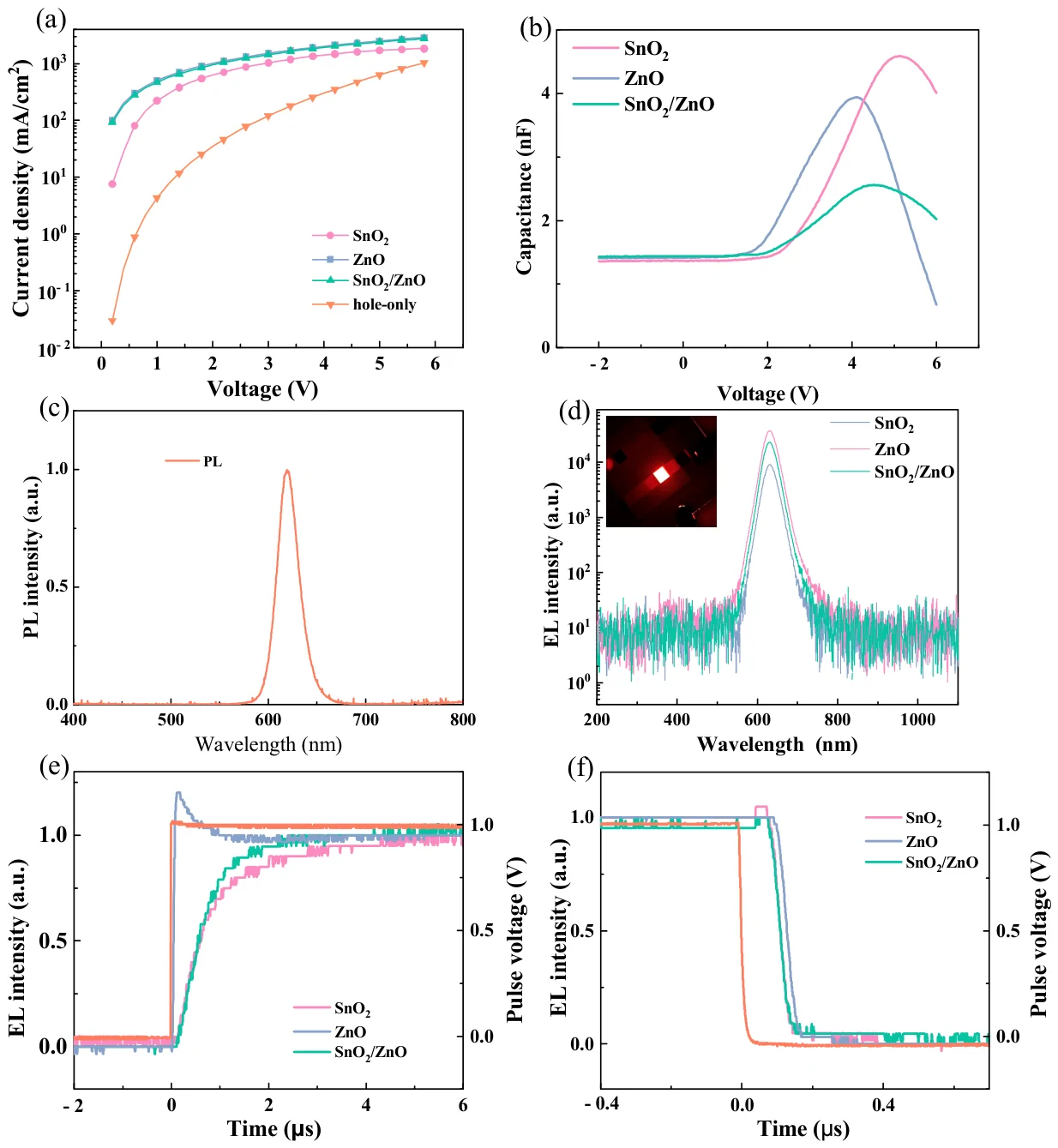
(**a**) *J-V* characteristics of the electron-only devices employing SnO_2_, ZnO, and SnO_2_/ZnO ETLs, together with the hole-only device; (**b**) *C–V* characteristics of the corresponding QLEDs; (**c**) Normalized PL spectrum of the CdSe/ZnS QD; (**d**) EL spectra of QLEDs employing SnO_2_, ZnO, and SnO_2_/ZnO ETLs at an applied voltage of 5.0 V, with an operating SnO_2_/ZnO QLED shown in the inset; (**e**) rising edges; and (**f**) falling edges of the TrEL responses. The orange curves in (**e**,**f**) represent the pulse-reference signals.

To further clarify the effects of the SnO_2_/ZnO bilayer ETL on electron transport and charge accumulation, electron-only devices and capacitance–voltage measurements were performed. Figure 5a compares the *J–V* characteristics of the electron-only devices with those of the hole-only reference. Over most of the voltage range, the ZnO and SnO_2_/ZnO-based electron-only devices showed higher current densities than the SnO_2_-based device, whereas the hole-only current remained substantially lower. These results show that the ZnO overlayer facilitates electron transport through the SnO_2_-based structure. To account for differences in transport-stack thickness, the electron-only currents were replotted against the nominal effective electric field, E=V/d, where *d* is the total oxide/QD/oxide thickness (Figure S5). These data compare the complete electron-transport pathways and do not isolate the contribution of the bottom ETL. The field-normalized results nevertheless show that the bilayer maintains efficient electron transport relative to SnO_2_. UV–visible absorption spectra of the processed SnO_2_ and ZnO films are shown in Figure S6.

Figure 5b shows the capacitance–voltage (*C–V*) characteristics of the three QLEDs. *C–V* measurements have been used to identify carrier-response and injection or accumulation-related transitions in QLEDs [36,37]. Because the measurements were performed at a single frequency, the capacitance response is used here only for comparison among the three devices. Before turn-on, all three devices showed a nearly constant capacitance of approximately 1.4 nF. These values mainly represent the geometric capacitance of the devices. After turn-on, the capacitance increased because of carrier injection and charge accumulation within the devices. The capacitance of the ZnO and SnO_2_/ZnO devices began to increase at a lower voltage than that of the SnO_2_ device. This trend is consistent with the turn-on voltages obtained from the *L–V* characteristics. Additional impedance–voltage and phase angle–voltage characteristics are presented in Figure S7. Compared with the SnO_2_ device, the SnO_2_/ZnO device showed a lower impedance and an earlier electrical transition, further supporting that the ZnO overlayer facilitates electron injection and transport.

Clear differences were also observed in the peak capacitance. The SnO_2_ device exhibited the highest peak capacitance of approximately 4.6 nF, followed by the ZnO device at approximately 3.9 nF. In contrast, the SnO_2_/ZnO device showed the lowest peak capacitance of approximately 2.5 nF. Since the three QLEDs employed identical hole-side layers, these differences are mainly related to the electron injection characteristics of the ETLs. The lower capacitance peak of the SnO_2_/ZnO device is consistent with reduced net charge accumulation and improved carrier balance.

Taken together, the electrical results show that the SnO_2_/ZnO bilayer ETL suppresses leakage current and maintains effective electron transport. Its lower capacitance response is also consistent with reduced net charge accumulation. The ZnO overlayer facilitates electron transport, while the underlying SnO_2_ layer moderates the electron supply. These effects promote more balanced electron and hole injection into the QD layer, thereby improving charge utilization and radiative recombination. This explains the enhanced current efficiency of the SnO_2_/ZnO QLED.

Following the analysis of electron transport and charge accumulation, the PL and EL spectra were examined to clarify the carrier recombination behavior. Figure 5c shows the normalized PL spectrum of the CdSe/ZnS QDs, with an emission peak at approximately 620 nm. As shown in Figure 5d, all three devices exhibited a dominant EL peak near 620 nm, confirming that the main emission originated from the QD layer. A weak band near 400 nm was observed only for the ZnO device under the same acquisition conditions. Similar high-energy emission has been assigned to CBP in a related QLED structure [38]. The voltage-dependent spectra show that this feature persisted for the ZnO device but was absent for the SnO_2_/ZnO device (Figure S8). This weak band is not used as direct evidence of electron overflow. Together with the electrical data, however, it is consistent with different carrier-confinement behavior in the two devices.

To further investigate the influence of different ETLs on the charge injection dynamics, the transient electroluminescence (TrEL) responses of the devices were measured. Figure 5e shows the rising edges of the TrEL responses. The signals were normalized only in intensity; the time axis was unchanged. The orange trace is the normalized pulse-reference signal, while each device was driven by a 0–5 V square-wave pulse at 1 kHz. Because the hole-side layers and measurement conditions were identical, the rising profiles allow a relative comparison of ETL-dependent carrier injection and transport. The SnO_2_ device exhibited the slowest EL rise. In comparison, the SnO_2_/ZnO device showed a faster response, indicating that the thin ZnO overlayer improves electron transport toward the QD layer. This result is consistent with the improved surface morphology and the electron-only device measurements. The ZnO device showed the fastest initial response and a clear overshoot. Such overshoot can arise from transient charge storage, trapping, or circuit response [39,40,41,42,43]. All devices were measured under identical conditions. The overshoot observed for ZnO but not for SnO_2_/ZnO is therefore consistent with different transient charge-storage behavior. We do not assign the overshoot to a single carrier process.

Figure 5f shows the falling edges of the TrEL responses. After removal of the voltage pulse, the EL signals of the three devices rapidly decayed toward the baseline and exhibited similar decay profiles. The similar decay profiles indicate no marked ETL-dependent difference during pulse turn-off under these conditions.

Together, these measurements clarify the role of the SnO_2_/ZnO bilayer. The ZnO overlayer smooths the SnO_2_ surface, consistent with the lower leakage current before turn-on, and maintains efficient electron transport through the SnO_2_-based structure. In the complete QLED, the lower current density than that of the ZnO device and the lower capacitance peak point to regulated injection and reduced net charge storage. The spectral and transient responses further support this picture.

**Figure 6:**
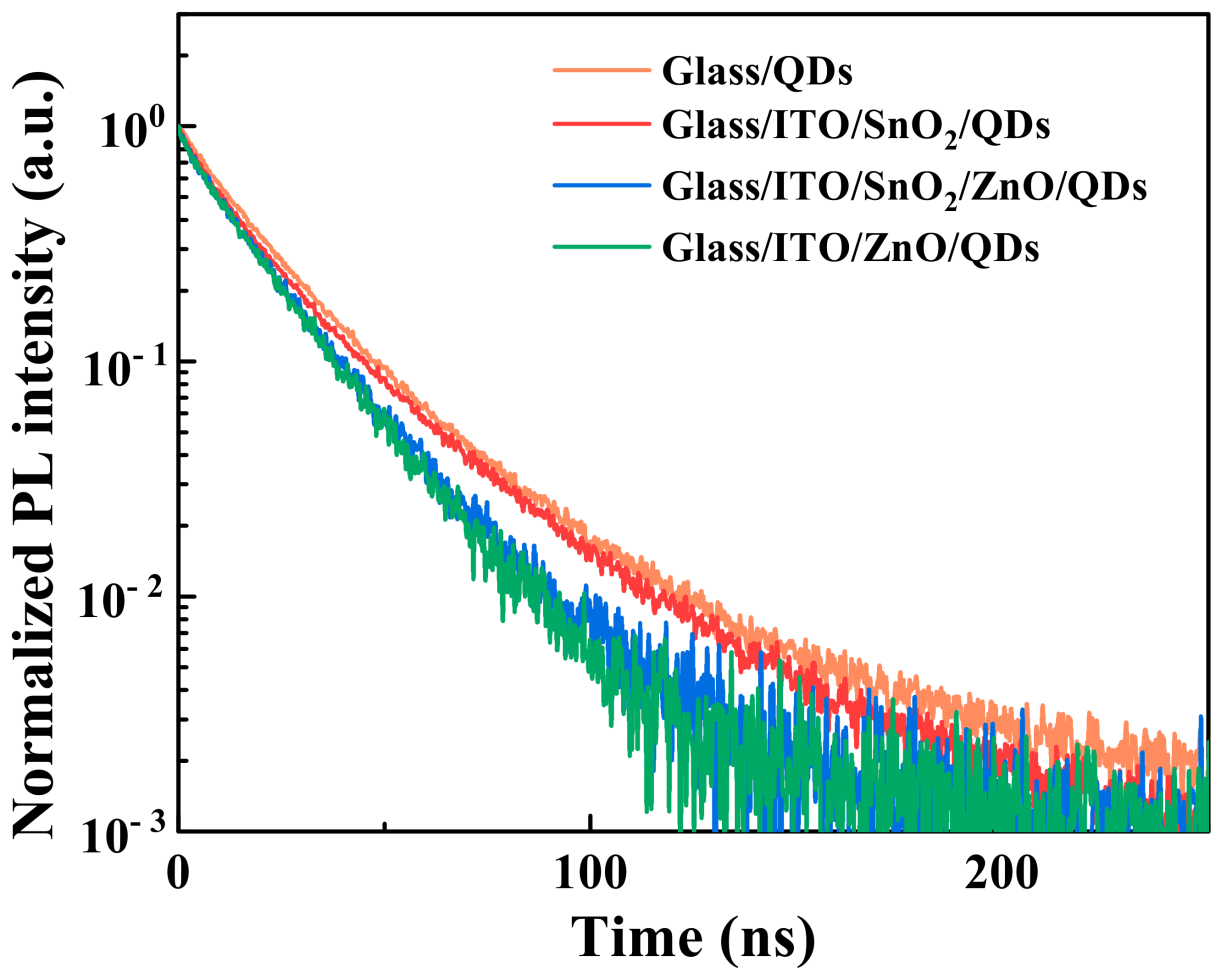
Normalized time-resolved PL decay curves of CdSe/ZnS QD films deposited on Glass, Glass/ITO/SnO_2_, Glass/ITO/SnO_2_/ZnO, and Glass/ITO/ZnO substrates.

To examine interfacial PL quenching, TRPL measurements were performed for QD films deposited on different substrates. As shown in Figure 6, the average PL lifetimes of Glass/QD, SnO_2_/QD, SnO_2_/ZnO/QD, and ZnO/QD were 19.85, 18.53, 16.36, and 15.64 ns, respectively. The longer lifetime of SnO_2_/QD relative to ZnO/QD agrees with the weaker interfacial quenching reported when SnO_2_ directly contacts the QDs [34]. The SnO_2_/ZnO sample showed an intermediate lifetime because the QDs contacted the thin ZnO overlayer. Thus, ZnO did not enhance the QD PL lifetime in the present structure. Even so, the SnO_2_/ZnO QLED showed the highest current efficiency. Its performance therefore reflects both the smoother QD film and the regulated electron-transport pathway, rather than PL lifetime alone. The decay curves were fitted with a biexponential function; the fitting parameters are listed in Table S2.

## 4. Conclusions

In summary, a SnO_2_/ZnO bilayer ETL was introduced to regulate electron injection in red CdSe/ZnS QLEDs. Depositing a thin ZnO overlayer reduced the RMS roughness of the SnO_2_ film from 1.79 to 1.07 nm and suppressed the leakage current. The bilayer ETL also enhanced electron transport compared with SnO_2_ while moderating the excessive electron injection associated with ZnO. As a result, the SnO_2_/ZnO device achieved a maximum current efficiency of 13.40 cd/A, representing improvements of 100% and 32.7% compared with the SnO_2_ and ZnO devices, respectively. The electron-only, impedance, and *C–V* measurements were consistent with effective electron transport and reduced net charge accumulation in the bilayer device. The EL and TrEL results further support controlled electron injection and reduced transient charge storage in the bilayer device. The TRPL data show that interfacial PL decay alone does not determine the efficiency enhancement; the regulated carrier-transport pathway also contributes. These results show that combining SnO_2_ and ZnO provides an effective approach to improve charge balance and device efficiency in QLEDs.

## Data Availability

The original contributions presented in this study are included in the article and Supplementary Materials. Further inquiries can be directed to the corresponding author.

## Supplementary Materials

The following supporting information can be downloaded at https://www.mdpi.com/article/10.3390/nano16161003/s1: Figure S1: AFM images of QD films deposited on SnO_2_, ZnO, and SnO_2_/ZnO ETLs; Figure S2: AFM step-height profiles of the SnO_2_, ZnO, SnO_2_/ZnO, and QD films; Figure S3: Surface morphology and device characteristics of the SnO_2_ and solvent-treated SnO_2_ controls; Figure S4: Device characteristics of QLEDs employing the reverse ZnO/SnO_2_ ETL; Figure S5: Current density–nominal effective electric field characteristics of the electron-only devices; Figure S6: UV–visible absorption spectra of the SnO_2_ and ZnO films; Figure S7: Impedance–voltage and phase-angle–voltage characteristics of QLEDs employing different ETLs; Figure S8: Voltage-dependent EL spectra of QLEDs employing different ETLs; Table S1: Performance parameters and statistical results of independently fabricated QLEDs; Table S2: Biexponential fitting parameters and average PL lifetimes of QD films deposited on different substrates.
