# Supplementary material for: A SnO2/ZnO Nanoparticle Bilayer Electron Transport Layer for Regulated Electron Injection in Quantum-Dot Light-Emitting Diodes
Source: Nanomaterials (Basel). 2026 Aug 15;16(16):1003. doi: 10.3390/nano16161003 (PMC13515732; doi:10.3390/nano16161003)
Supplement: Supplementary file 1 [file nanomaterials-16-01003-s001.zip › nanomaterials-4493004-supplementary.pdf]

# A $SnO_2$/ZnO Nanoparticle Bilayer Electron Transport Layer for Regulated Electron Injection in Quantum-Dot Light-Emitting Diodes

**Yuechao Wang [1,*], Xiongqiang Ma [2], Ruirong Wang [1] and Junsheng Zhang [1]**

1 Department of Electronic Engineering, Taiyuan Institute of Technology, Taiyuan 030008, China; wangrr@tit.edu.cn (R.W.); zhangjs@tit.edu.cn (J.Z.)
2 Department of Mining Engineering, Shanxi Institute of Energy, Taiyuan 030006, China; maxq@sxie.edu.cn
* Correspondence: wangyuechao@tit.edu.cn

Figure S1 shows the AFM images of the QD films deposited on the different ETLs. The QD films on $SnO_2$, ZnO, and $SnO_2$/ZnO exhibited RMS roughnesses of 1.80, 1.32, and 1.41 nm, respectively.

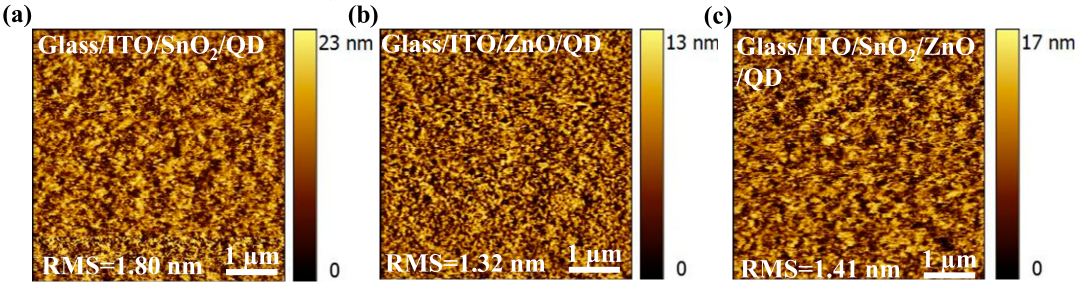


**Figure S1.** AFM images of QD films deposited on (a) ITO/$SnO_2$, (b) ITO/ZnO, and (c) ITO/$SnO_2$/ZnO substrates.

Figure S2 shows the AFM step-height profiles used to determine the film thicknesses. The films were prepared under the same conditions used for the QLEDs. The thickness increased from approximately 40 nm for $SnO_2$ to 50 nm for $SnO_2$/ZnO. This increase is consistent with the retention of the $SnO_2$ layer and the deposition of a thin ZnO overlayer.

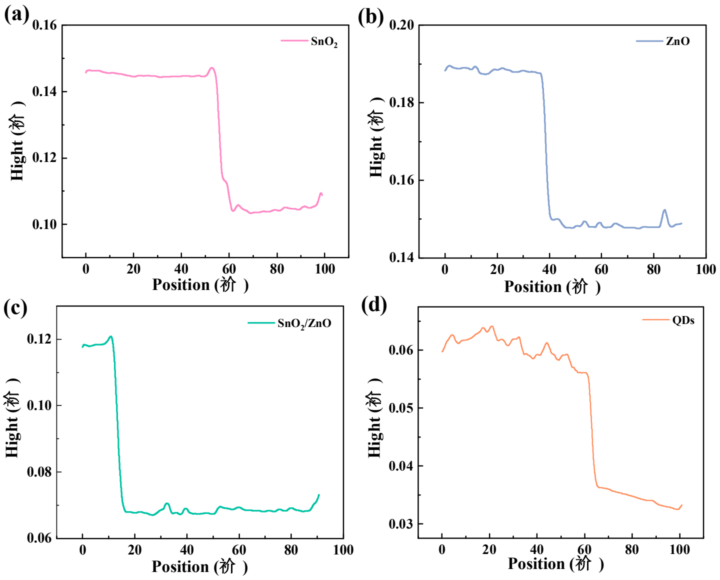


**Figure S2.** AFM step-height profiles of the (a) $SnO_2$, (b) ZnO, (c) $SnO_2$/ZnO, and (d) QD films. The corresponding film thicknesses were approximately 40, 40, 50, and 20 nm, respectively.

Figure S3 shows the surface morphology and device characteristics of the process-matched $SnO_2$–solvent control. Its RMS roughness was 1.64 nm, close to 1.79 nm for untreated $SnO_2$. The two devices also showed similar *J–V*, *L–V*, and *CE–J* characteristics. The solvent treatment and second annealing therefore had little effect on the $SnO_2$ film or device performance.

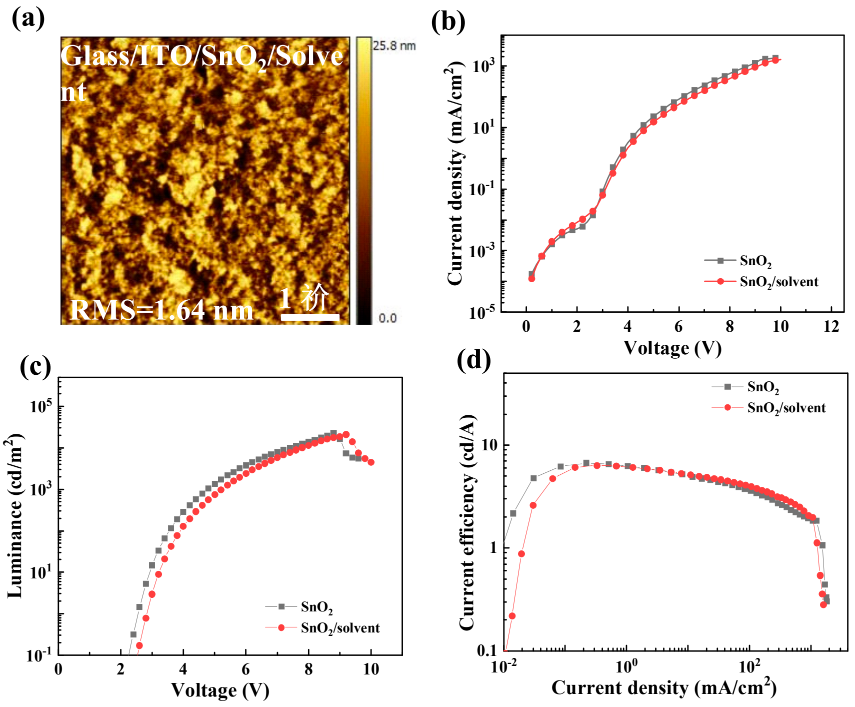


**Figure S3.** (a) AFM image of the $SnO_2$ film after solvent treatment and annealing at 120 °C for 30 min. Comparison of the (b) *J–V*, (c) *L–V*, and (d) *CE–J* characteristics of the untreated $SnO_2$ and process-matched $SnO_2$–solvent QLEDs.

Figure S4 compares the device characteristics of the reverse-order $ZnO/SnO_2$ QLED with those of the single-layer ZnO QLED. The reverse-order device was prepared using 20 mg/mL ZnO and 7 mg/mL $SnO_2$. Its *J–V*, *L–V*, and *CE–J* characteristics were close to those of the single-layer ZnO device, and no efficiency improvement was observed. This comparison shows that the bilayer effect depends on the oxide sequence.

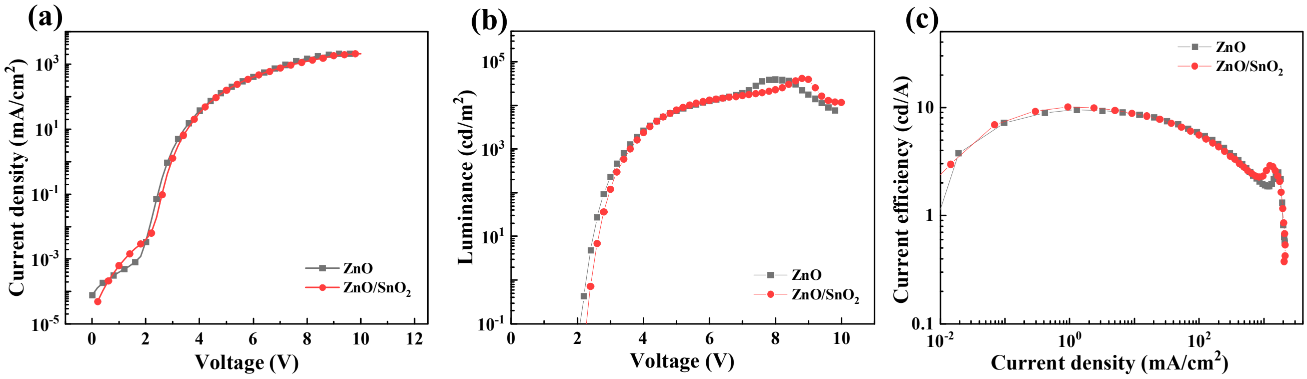


**Figure S4.** Comparison of the (a) *J–V*, (b) *L–V*, and (c) *CE–J* characteristics of the single-layer ZnO and reverse-order $ZnO/SnO_2$ QLEDs.

**Table S1.** Individual and statistical performance parameters of QLEDs employing different ETLs.

| ETL | $L_{max}$ (cd/m²)[a)] | $V_T$ (V)[b)] | $CE_{max}$ (cd/A)[c)] | $J_{leak}$ at 1.8 V (mA/cm²)[d)] | $CE_{1000}$ (cd/A)[e)] | $EQE_{max}$ (%)[f)] |
|---|---|---|---|---|---|---|
| $SnO_2$ | 22,770 | 2.425 | 6.704 | 0.004580 | 4.791 | 4.490 |
| $SnO_2$ | 19,710 | 2.300 | 6.233 | 0.005768 | 5.309 | 4.175 |
| $SnO_2$ | 18,880 | 2.280 | 6.226 | 0.005030 | 5.451 | 4.171 |
| $SnO_2$ | 19,030 | 2.443 | 6.165 | 0.000612 | 4.836 | 4.130 |
| Mean ± SD | 20,098 ± 1,818 | 2.362 ± 0.084 | 6.332 ± 0.250 | 0.003998 ± 0.002309 | 5.097 ± 0.333 | 4.241 ± 0.167 |
| ZnO | 39,180 | 2.231 | 10.058 | 0.000812 | 8.626 | 6.738 |
| ZnO | 36,210 | 2.229 | 9.590 | 0.000724 | 7.998 | 6.424 |
| ZnO | 39,500 | 2.200 | 10.983 | 0.003411 | 8.870 | 7.357 |
| ZnO | 36,220 | 2.220 | 10.657 | 0.001863 | 8.671 | 7.139 |
| Mean ± SD | 37,778 ± 1,809 | 2.220 ± 0.014 | 10.322 ± 0.620 | 0.001703 ± 0.001251 | 8.541 ± 0.377 | 6.914 ± 0.416 |
| $SnO_2$/ZnO | 33,210 | 2.267 | 13.398 | 0.001370 | 9.395 | 8.975 |
| $SnO_2$/ZnO | 38,170 | 2.202 | 13.952 | 0.002573 | 10.693 | 9.346 |
| $SnO_2$/ZnO | 34,470 | 2.204 | 13.211 | 0.003986 | 9.893 | 8.849 |
| $SnO_2$/ZnO | 36,170 | 2.120 | 13.380 | 0.006123 | 9.999 | 8.963 |
| Mean ± SD | 35,505 ± 2,151 | 2.198 ± 0.060 | 13.485 ± 0.322 | 0.003513 ± 0.002042 | 9.995 ± 0.535 | 9.033 ± 0.216 |

[a)] $L_{max}$ is the maximum luminance of the device; [b)] $V_T$ is the turn-on voltage at a luminance of 0.1 cd/m²; [c)] $CE_{max}$ is the maximum current efficiency of the device; [d)] $J_{leak}$ is the leakage current density at 1.8 V; [e)] $CE_{1000}$ is the current efficiency at 1000 cd/m²; [f)] $EQE_{max}$ is the maximum external quantum efficiency.

Figure S5 shows the electron-only current densities replotted against the nominal effective electric field, $E=V/d$, to account for the different transport-stack thicknesses. Here, $d$ is the total oxide/QD/oxide thickness. The corresponding values were approximately 100 nm for the $SnO_2$ and ZnO devices and 120 nm for the $SnO_2$/ZnO device. At the same field, the current density followed the order $SnO_2$/ZnO > ZnO > $SnO_2$. The bilayer therefore maintains efficient electron transport relative to $SnO_2$.

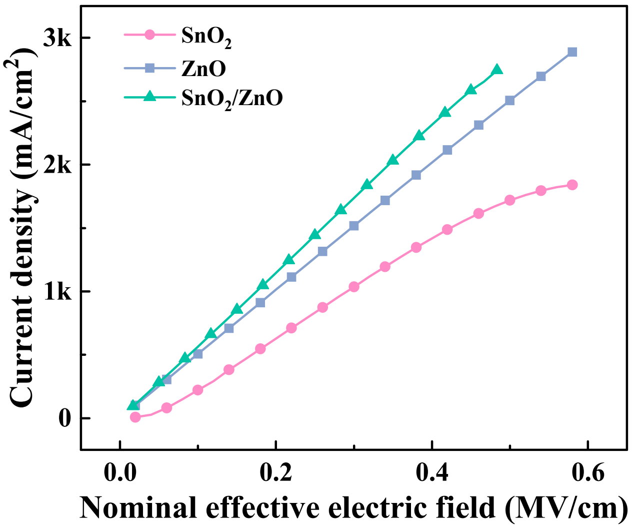


**Figure S5.** Current density–nominal effective electric field characteristics of the electron-only devices employing $SnO_2$, ZnO, and $SnO_2$/ZnO ETLs. The nominal effective electric field was calculated using the total thickness of the oxide/QD/oxide stack.

Figure S6 shows the UV–visible absorption spectra of the $SnO_2$ and ZnO films. Both films showed low absorption in the visible region and increased absorption toward the ultraviolet region, consistent with their wide-bandgap oxide characteristics.

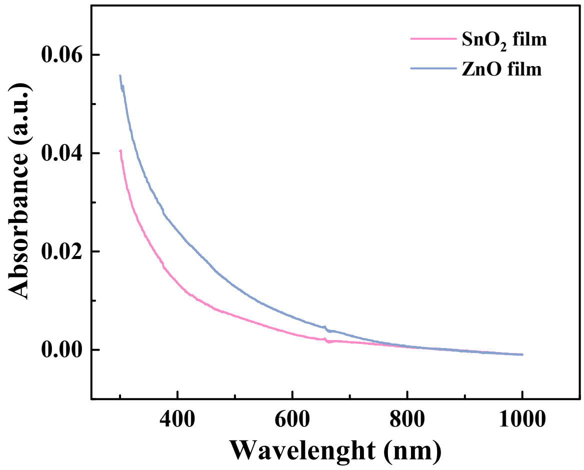


**Figure S6.** UV–visible absorption spectra of $SnO_2$ and ZnO films prepared under the same deposition conditions as those used in the QLEDs.

Figure S7 compares the impedance and phase-angle responses of the three QLEDs. Before turn-on, the $SnO_2$ device showed the highest impedance. The ZnO overlayer lowered the impedance of the $SnO_2$-based device and shifted the electrical transition to a lower voltage. The transition nevertheless occurred after that of the ZnO device. The phase-angle changes appeared over the same voltage range.

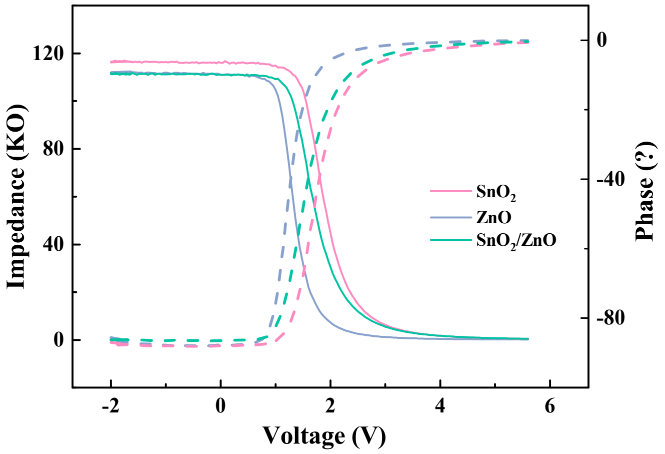


**Figure S7.** Impedance–voltage characteristics (solid lines, left y-axis) and phase angle–voltage characteristics (dashed lines, right y-axis) of QLEDs employing $SnO_2$, ZnO, and $SnO_2$/ZnO electron transport layers.

Figure S8 shows the voltage-dependent EL spectra of the three QLEDs. The main QD emission remained near 620 nm from 3.0 to 5.0 V. A weak band near 400 nm was observed for the ZnO device and persisted with increasing voltage. A similar band was assigned to CBP emission in a related QLED structure [36]. No corresponding feature was detected for the $SnO_2$/ZnO device. Because this feature is weak, it is not used as independent evidence of electron overflow.

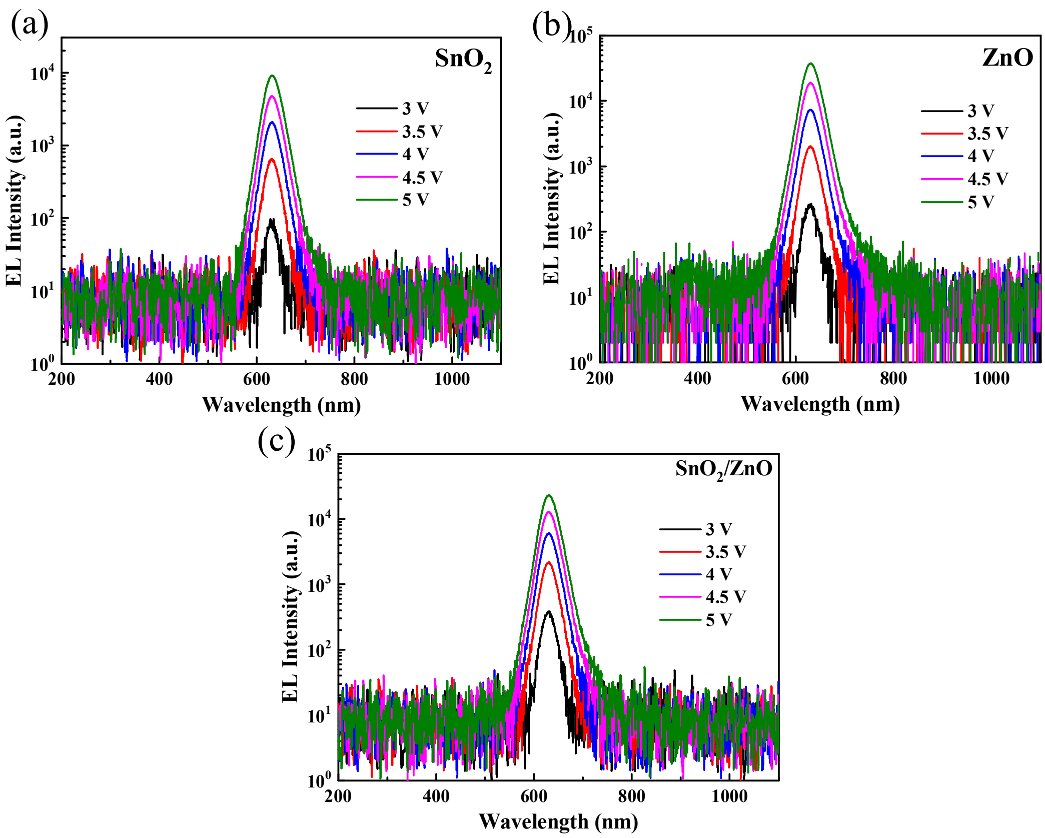


**Figure S8.** Voltage-dependent electroluminescence spectra of QLEDs employing (a) $SnO_2$, (b) ZnO, and (c) $SnO_2$/ZnO electron transport layers, recorded at applied voltages of 3.0, 3.5, 4.0, 4.5, and 5.0 V.

**Table S2.** Biexponential fitting parameters and average PL lifetimes of QD films on different substrates.

| Sample | $A_1$ | $\tau_1$ (ns) | $A_2$ | $\tau_2$ (ns) | $\tau_{avg}$ (ns) |
|---|---|---|---|---|---|
| Glass/QD | 0.513 | 11.95 | 0.487 | 28.17 | 19.85 |
| Glass/ITO/$SnO_2$/QD | 0.425 | 9.01 | 0.550 | 25.89 | 18.53 |
| Glass/ITO/$SnO_2$/ZnO/QD | 0.187 | 4.79 | 0.774 | 19.16 | 16.36 |
| Glass/ITO/ZnO/QD | 0.145 | 2.95 | 0.832 | 17.85 | 15.64 |
